# Participant experiences of change in mindfulness-based stress reduction for anxiety disorders

**DOI:** 10.1080/17482631.2020.1776094

**Published:** 2020-06-16

**Authors:** Elisabeth Schanche, Jon Vøllestad, Per-Einar Binder, Aslak Hjeltnes, Ingrid Dundas, Geir Høstmark Nielsen

**Affiliations:** a Department of Clinical Psychology, University of Bergen, Bergen, Norway; b Solli District Psychiatric Centre (DPS), Nesttun, Norway

**Keywords:** Qualitative Research Methods, anxiety, mindfulness, mindfulness-Based Stress Reduction

## Abstract

**Aim:**

To explore experiences of change among participants in a randomized clinical trial of mindfulness-based stress reduction (MBSR) for anxiety disorders.

**Method:**

Semi-structured interviews were conducted to explore the subjective experiences of change for individuals with anxiety disorders after a course in MBSR. Interviews were analysed employing hermeneutic-phenomenological thematic analysis.

**Results:**

Five main themes were identified: 1) Something useful to do when anxiety appears, 2) Feeling more at ease, 3) Doing things my anxiety wouldn’t let me, 4) Meeting what is there, and 5) Better—but not there yet. Most participants used what they had learned for instrumental purposes, and described relief from anxiety and an increased sense of personal agency. A few reported more radical acceptance of anxiety, as well as increased self-compassion.

**Conclusion:**

Participants of MBSR both describe mindfulness as a tool to “fix” anxiety and as bringing about more fundamental change towards acceptance of their anxiety. The complexity of reported change corresponds with better handling of areas representing known transdiagnostic features of anxiety disorder, such as dysfunctional cognitive processes (including attentional biases), emotional dysregulation, avoidance behaviours, and maladaptive self-relatedness. This supports MBSR as a transdiagnostic approach to the treatment of anxiety disorders.

Mindfulness-based stress reduction (MBSR; Kabat-Zinn, 1990) is a group-based psychoeducational programme shown to be effective in coping with stress in a wide range of populations, including individuals with anxiety and anxiety disorders (Bamber & Morpeth, 2019; De Vibe, Bjørndal, Fattah, Dyrdal, Halland, & Tanner-Smith, 2017; Wharton & Kanas, 2019). Mindfulness encompasses self-regulation of attention to monitor present-moment experience, and the concurrent attitudinal qualities of acceptance, openness and non-judgement (Bishop et al., 2004; Creswell et al., 2019). Increased mindful awareness is assumed to facilitate a non-reactive and more flexible way of processing potentially distressing thoughts, feelings and bodily sensations (Feldman & Kuyken, 2019; Van der Velden et al., 2015; Williams, 2010). Moreover, therapeutic work is embedded in a framework of de-emphasizing the removal of unpleasant thoughts and feelings. Instead, the focus is on the possibility of living a meaningful life, regardless of anxiety-related discomfort (Eifert & Forsyth, 2005).

Despite a growing evidence base for MBSR in the treatment of anxiety, it is unclear which elements of MBSR have an impact on anxiety (Wharton & Kanas, 2019). Studies on possible mechanisms of change within mindfulness-based interventions (MBIs) highlight the complexity of the individualized change processes resulting from participation in these interventions (Grabovac et al., 2011; Van der Velden et al., 2015). According to Grabovac et al. (2011), no existing model appears to be sufficiently comprehensive in describing the details of how people change in MBIs. Thus, there is a need for a broad variety of methodological approaches in order to capture the complexity and heterogeneity of change processes within and between individuals participating in various mindfulness-based interventions. Qualitative studies have the potential of systematically investigating the lived experiences of clients and therapists in psychotherapy (Elliott, 2010; McLeod, 2011), and may be particularly well suited to exploring the complex process of change in MBIs (Malpass et al., 2012; Wyatt et al., 2014).

To date, two meta-syntheses conducted by Malpass et al. (2012) and Wyatt et al. (2014) have examined the existing qualitative research on mindfulness-based interventions. Both these summaries indicate a need for more qualitative studies of MBSR for clients with anxiety disorders. To our knowledge, only two studies have investigated MBSR for this population (A. Hjeltnes et al., 2018a, 2018b). Hjeltnes (2018b) compared how improved and less-improved participants with social anxiety disorder experienced the MBSR program, and examined how improved participants experienced their own process of change after the intervention. Hjeltnes and collegues explored the experiences of participants in a study of MBSR for academic performance anxiety (A. Hjeltnes et al., 2015). There is thus a lack of qualitative studies on how individuals with different anxiety disorders who have participated in MBSR experience and use mindfulness-based interventions in their everyday life.

The present study is part of a randomized controlled trial investigating the effects of Mindfulness Based Stress Reduction (MBSR) for a sample of Norwegian clients with heterogeneous anxiety disorders (Vøllestad et al., 2011). The aim of the present study was to explore the participants’ experiences of change after the MBSR program. We wanted to investigate how participants made sense of the MBSR program, how they made use of what they might have learned during the course in their everyday life, as well as their experienced benefit of applying the skills they had learned. In this article, we investigated the following research question: Which changes did participants experience after taking part in an eight-week MBSR program for anxiety disorders?

## Methods

### Setting and participants

The study was carried out in the context of a randomized controlled trial of MBSR for clients with heterogeneous anxiety disorders (for a detailed description of the larger study sample, please see Vøllestad et al., 2012). Participants had volunteered for the study on the basis of a newspaper advertisement and were assessed for eligibility by a brief phone screening and a subsequent comprehensive diagnostic assessment. For the diagnostic assessment, we used the Norwegian version of the Mini International Neuropsychiatric Interview [MINI] (Sheehan et al., 1998; Norwegian version Leiknes et al., 2007). Participants were eligible for inclusion if they were 1) between 18 and 65 years of age, and 2) fulfilled diagnostic criteria for either panic disorder with or without agoraphobia, social phobia, or generalized anxiety disorder according to the assessment. Exclusion criteria were: 1) suicidality, 2) substance abuse and/or dependence, 3) severe mental disorder (psychosis or bipolar disorder), 4) other Axis I disorders as primary diagnosis, 5) use of anxiolytics, 6) deficits in impulse control as assessed by the MINI module for antisocial personality disorder, and 7) other concurrent treatment. These criteria allowed for the presence of comorbid Axis I symptomatology, provided that patients had anxiety disorder as a primary diagnosis. Concurrent SSRI or MAOI use was allowed provided a stable dosage > 3 months and willingness not to alter dosage. In the larger study sample, 76 eligible participants were randomized to either MBSR or waitlist condition (WLC). Waitlist participants received treatment after eight weeks. Thirty-nine patients were in the MBSR condition. Of these, 1 dropped out before the intervention, and 7 dropped out during the intervention due to scheduling conflicts (1), practice requirements (3), symptoms exacerbated (1), and unknown (3).

The sub-sample used for the present study consisted of 16 randomly chosen participants, seven men and nine women from the MBSR condition. Age ranged from 28 to 64 years, with a mean age of 42.7 (SD = 11.18). Five were single or divorced, 10 married or cohabiting. Seven worked full time, 3 part-time, 2 were students, and 4 were on social benefits. Years of education ranged from 11–25 years with a mean of 16.9 years (SD = 3.63). Six participants had a primary diagnosis of panic disorder, 5 had a primary diagnosis of social anxiety disorder, and 5 had a primary diagnosis of generalized anxiety disorder. Eight (50%) used psychofaramcological medication. The duration of anxiety as reported by the interviewees ranged from three to 30 years, with a mean of 11.8 years (SD = 8.41).

### Intervention

The MBSR intervention offered in the present study followed the standard protocol, described by Kabat-Zinn (1990). MBSR is a manualized, group-based training program designed to enable patients to learn skills that reduce stress and cultivate ways of relating more flexibly to own experiences. MBSR rests on the assumption that bringing non-reactive awareness to situations of distress will enable more adaptive responding. The program takes the form of eight two hour weekly group sessions, an all-day silent retreat, and individual daily homework in between sessions. The intervention was delivered in a University setting with two MBSR groups of 15–20 patients in each group. Each session, participants were introduced to a theme relevant for understanding how habitual modes of thinking, feeling, and behaving that contribute to stress and to various formal and informal practices that aim to facilitate mindful awareness of thoughts, emotions and bodily sensations. Throughout, participants were invited to share their experiences with the practice with the instructors and other members of the group. An aim of MBSR instructors is to convey the course themes through interactive inquiry and didactic teaching, and through embodying the attitudinal components of openness, curiosity, and compassion. There was an emphasis on the non-instrumental: the work was to stay present with whatever came up, whether one liked it or not. Goals were not formulated in terms of getting rid of symptoms, but rather in terms of relating differently to discomfort in everyday life. As the program proceeded, participants were increasingly invited to be mindful of their own adverse experiences, including thoughts, feelings and body sensations situations of distress. In line with the MBSR manual (Kabat-Zinn, 1990), the participants were encouraged to practice at home between group-sessions. To aid their home practice, they were given access to recordings of formal mindfulness practices made by the instructor of the MBSR group. The recordings were based on a translation of recording used in the standard manual of Kabat-Zinn (1990).

### Procedure

At post-treatment assessment, participants were asked for consent to be contacted for a follow-up interview. They were told that we wanted to learn more about their subjective experiences of participating in the MBSR course and changes that might have occurred during or after the course. The information given was the same for all participants. We also informed them that we would not interview all, but randomly select who would be contacted for interviews. All treatment completers consented, and 16 participants were randomly chosen for interviews. The randomization process was carried out by a colleague not involved in the project, using the random number generation function (RAND) of Microsoft Excel (Microsoft Inc, Redmond, WA). Further recruitment of participants was possible during analysis, but we considered the sample sufficient for establishing and illuminating the themes in this study. The interviews were conducted within one month of completion of their MBSR course. Three members of the research team carried out the interviews. The researchers who performed the interviews had not met any of the participants prior to the interview situation. Interviews were transcribed by a graduate student in clinical psychology who was not part of the research group.

### Data collection method

We conducted this qualitative investigation in line with the principles of hermeneutic phenomenology, aiming for maximally rich and valid representations of the participants’ lived experience (Binder et al., 2012). A phenomenological approach focuses on understanding the lived experience of an individual and his or her perceptions of events or objects (Van Manen, 2016). From a hermeneutical perspective, meaning is seen as fully dependent on a process of interpretation and takes into account that one cannot understand the participants directly, but only through the researchers’ own perspective (Finlay, 2003).

### Interviews

The interviews were semi-structured and aimed to facilitate an explorative dialogue of participants’ experience of outcome after the MBSR course. The interviews lasted from 42 to 82 min (mean = 66 min), and were audio‐recorded and subsequently transcribed verbatim.

An interview schedule with three general questions was used. The questions invited participants to talk about (1) their backgrounds and what made them sign up for the course, (2) their experience of participating in the program, and (3) changes that might have occurred in their life during or after the course. The aim was to increase the accuracy of our understanding during the interview by asking for concrete examples whenever possible, and “reflecting back” our understandings to the participants for him or her to correct and elaborate. As the focus of the current study was experiences related to change, most emphasis was put on accounts elicited by the third general question and its corresponding probes.

### Data analysis

The data analyses of the transcribed interviews were conducted using a team-based explorative–reflective thematic analysis (Binder et al., 2012). By comparing the individual interviews, we sought to identify patterns of meaning in how the participants experienced change in the MBSR program. A “meaning pattern” can be defined as a condensed summary of the units of relevance for a particular research topic that may be identified when comparing the experiences of several participants (Binder et al., 2012). The sequence of the analysis can be described as follows (see Table 1): (1) Each transcribed interview was read thoroughly by five of the co-authors to capture the first impression of aspects of the text relevant to the research question. (2) The second author examined every part of the text relevant to the research question, and each part of the text expressing different aspects of the participants’ experiences of participating in the MBSR intervention was marked and named as meaning units. (3) Looking at the meaning units across interviews, the second author identified and condensed meaning codes across different participants, staying as close to the informants’ use of language as possible. (4) Following this first organization of the data, the second author and three co-authors summarized and edited the meaning within each of the coded groups of text fragments. The research team then developed conceptions and overall descriptions of meaning patterns and themes, reflecting what was identified as the most important aspects of the participants’ experiences. (5) Preliminary results of the analysis were then presented to the last author, who acted as a critical auditor for the thematic structure and wording of the themes and sub-themes. (6) The themes were finally formulated, revised and agreed upon by the research team. For a summary and elaboration of each step in the analytic process, see Table I.

**Table I.** 

Research principles	Description of the research process
1. Familiarization with the data:	Five of the co-authors read the transcribed material to obtain a basic sense of the experiences described by the participants. They discussed these impressions to establish a basic sense of both the heterogeneity and the homogeneity of the participants’ experiences, and to increase reflexive awareness of their immediate responses to the material.
2. Searching for aspects of the transcribed material relevant to the research question:	Examining the transcribed material, the second author identified separable meaning units that represented different aspects of the participants’ experiences. He would here look at how the participants described their experiences of taking the mindfulness-based stress reduction (MBSR) program in relation to changes that might have occurred in their life during or after the course.
3. Coding themes:	The second author developed “meaning codes” for those units, which are concepts or keywords attached to a text segment in order to permit its later retrieval. The text where then edited in accordance with those codes into coded groups of text with the technical assistance of Nvivo 8 software. For example, the participants’ descriptions of experiencing increased calm and less stress were given the code “Less stress”.
4. Reviewing and summarizing themes and sub-themes:	The second author then met with three of the other co-authors who had read each interview thoroughly. This team of researchers summarized and edited the meaning within each of the coded groups of text fragments. They then developed conceptions and overall descriptions of meaning patterns (main themes) and sub-themes, reflecting their interpretation of what were the most important aspects of the participants’ experiences.
5. Critical auditing:	The last author, who was not familiar with the concepts of mindfulness and the MBSR intervention, had a leading role in critically auditing the identification of meaning patterns (themes).
6. Forming a consensus on themes:	The themes were finally formulated, revised and agreed upon by the research team.

### Researchers

The interviews were conducted by the first, third and fifth author, none of which acted as MBSR instructors in the study. These researchers had not met any of the participants prior to the interview situation. The interviews were recorded and transcribed verbatim for analysis. The second author led the intervention group and was in charge of the data analytic procedure. All authors, with exception of the last, are clinical psychologists with a personal mindfulness practice, and are trained as instructors in MBSR and/or MBCT.

### Ethics

The study was approved by the Regional Committee for Medical Research Ethics in

Norway. It was also reported to the Norwegian Centre for Research Data. Participants gave an informed written consent, could withdraw from the study at any time without stating any reason, and did not receive any financial reimbursement.

## Results

Five main themes were identified on the basis of the accounts of participants about their experiences of change after the MBSR program. These were named as follows: 1) Something useful to do when anxiety appears, 2) Feeling more at ease, 3) Doing things my anxiety wouldn’t let me, 4) Meeting what is there, and 5) Better—but not there yet. In presenting the findings, “all participants” implies all 16 participants, “most participants” implies 13–15 participants, “many” implies 9–12, and “some” implies 5–8 participants.

### Something useful to do when anxiety appears

#### Concrete focus on bodily sensations or practical tasks

Most participants expressed that the course had provided them with strategies or tools that could be employed in stressful or anxiety-provoking situations. Many participants found it easier to bring their attention away from their own distressing inner narratives and images, and onto a concrete focus, either in the form of awareness of the body (including the breath), the external situation, or the task at hand. One man described:

I have experienced that it is a way to bring your awareness away from thoughts onto something concrete. You always have access to both the breath and bodily sensations. So by using them as an anchoring point, you have the option to bring your awareness to them at any time. And then bring forth the calm that you have experienced during meditation.

This participant experienced that this concrete focus helped him to remain present in situations that he would otherwise be prone to leave due to his agoraphobic fears. Another participant described how deliberately taking control over her breath and avoiding shallow breathing reduced her anxiety:

If I get into stressful situations, I start at once, before I become defensive and start thinking ‘no, now I’m scared’. I think ‘breathe out and in’, breathing all the way down instead of just up here, so I start to think about that right away, instead of thinking ‘oh, I’m afraid of this, I can’t do this’.

Some participants also described taking time-out to do mindful movements, sitting meditation, or awareness of washing hands to get a sense of grounding that would decrease their level of anxiety. A woman explained:

When I get stressed, I tend to breathe up here, and the thoughts flit around up here, and then when I go in to wash, and meditate on washing my fingers, then you’re supposed to concentrate strongly on that, and then all other thoughts disappear, and the breath enters the belly, and you sense something falling to rest. And then in a way you’re in touch with the ground again. So that I think was very lovely.

#### Letting go of negative thinking

Some participants described the ability to let go of negative and self-critical thinking or rumination as a benefit they had received from the MBSR course. Among these, a subset described a reduction in the frequency of difficult thoughts, as illustrated by the participant who stated that “I notice that I manage to get some time off from my thoughts.” Others emphasized to a greater extent being able to allow the thoughts to come and go:

I am able to a greater extent to let circles of negative thoughts disappear. I can, to a greater degree, just let go, and not get stuck in a loop of self-reinforcing negative thoughts either about myself or about others, and thoughts about others’ thoughts about me, and so on. Which for me has been a large problem, worrying about pointless issues that don’t really help me make decisions, stuff that doesn’t help me solve things, but that nevertheless has occupied an unproportionally large part of my time and attention.

The participants’ freedom from these thoughts gave them a more solid sense of reality, as opposed to the imaginary or exaggerated character of their worries. A few participants also noted that it represented a break with their habitual tendency of wanting to analyse their present state of mind, in order to find explanations and solutions. One woman found letting go of the tendency to analyse her thoughts unfamiliar and “unnatural”, but believed that it would be helpful in the long run. Another commented that this approach was different from her earlier experiences in psychotherapy, which she saw as more focused on analysing her experience and finding the causes for her anxiety.

These two set of skills, focusing on the concrete and letting go of thoughts, are intertwined in most accounts. That is, the disengagement of thoughts seems to be made possible by having somewhere else to focus, namely on the felt sense of the body or breath or one’s surroundings.

### Feeling more at ease

Many participants noted less bodily discomfort, fewer anxiety attacks and reduced ongoing tension as a result of the course. Some reported that they recognized a decrease in the frequency and intensity of anxiety or worrisome thoughts; others used wordings like becoming “calmer”, “more relaxed”, or “more harmonious”. Some participants also conveyed an experience of having less stress in general in their lives, due to a greater sense of ease and an increased sense of coping with everyday challenges. One man explained:

It gives you increased calm and harmony, plain and simple. Especially by getting out of thought loops that run on and on, which contribute to creating stress over time. So by getting out of those, you prevent stress.

A woman described the time after the course as particularly challenging due to a number of life events, and said that she would ordinarily be very stressed out and “run down” after such a time. But she felt that she had been able to do things differently, in the sense of reminding herself that there was no rush and by making clearer priorities about the importance of tasks. This is in line with what was reported by several other participants, in that there seemed to have been a positive change in perceptions of one’s own ability to handle challenges—both those pertaining to anxiety-laden situations and to life stressors in general.

### Doing things my anxiety wouldn’t let me

#### Going places or doing things that used to be avoided

Half of the participants talked about how the course had helped them pursue activities or seek out situations that their anxiety had prevented them from taking part in. These included activities like shopping, travelling, attending football matches and social gatherings. Some described doing new things despite anxiety being present, by using the opportunities to shift their focus onto concrete aspects of their experience as described above. For instance, one woman described how she now was able to go out for coffee:

Just going to a café was a pain before. Sitting at a table, if I was sitting like this it was okay but if I was sitting on a wooden chair then I couldn’t concentrate at all, I’d just be preoccupied with not falling off the chair. But now that’s a lot better, because I recognize “breathe … breathe … breathe.

The final part of this statement points to a recurring theme in relation to the acquisition of skills, that is, the increased sense of control or predictability provided by new ways of coping.

#### Doing things differently in relationships

Most participants conveyed an experience of doing things differently in their relationships with others. This involved being able to speak one’s mind and communicating more clearly. Several mentioned handling demands from others in the workplace with greater assertiveness. Others reported increased awareness of their own needs and boundaries including a sense of what was right or wrong for them. This awareness helped them to take better care of themselves and stand up for themselves. One woman told the interviewer that:

I sense that if there’s something disagreeing in me, in the sense that ‘No, I really don’t want this’, then I’ve become more aware that ‘okay, I don’t really want this, and then I’m not supposed to do it now’, in a way … like, trying to choose something that gives me something in return.

In this and other accounts, a greater sense of agency was clearly present.

The second major theme in relation to others was an increased ability to listen or an increased awareness of the struggle of paying thorough attention to others. Some of the exercises during the course were aimed at practicing listening, and one participant described that as bringing about insight for him:

That was an a-ha experience as well. I listened much better to what the other was saying than what is usually the case.

Several mentioned that they now focused less on what they themselves were about to say and more on what their conversation partner was actually saying. It seemed from their narratives that as their anxiety decreased, so did their pre-occupation with themselves. Others described that they still struggled with paying attention when others spoke, but that they had gained an increased awareness of this tendency.

### Meeting what is there

#### Stopping and noticing

Some of the participants described an increased ability to pause when things became difficult. This enabled them to notice negative thoughts or tension in the body, and to relax and proceed more slowly instead of reacting spontaneously in the ways they had previously done. One participant noted: “I still get stressed. But what I’ve learned is to stop when I sense my breath is up here. Then it’s: ‘okay’. I haven’t done that before. I’ve just kept on going.” Another stated that:

What I learned most from [the intervention] was this [procedure of] ‘stop, and notice where you are, and what is making you uneasy’. Yes. I notice now, if I start to increase my stress level, at least I manage to stop and take a step back and look at the situation.

Becoming familiar with their own reactions seemed to help them to be less governed by the anxiety:

I have a greater capacity to use some seconds to try to put into words and be more aware of what I’m experiencing [and] why. And if I can’t find a reason, at least postponing the exit or flight reaction.

Noticing and discerning different thoughts, feelings and bodily sensations was also noted by some participants. One man described how the course had helped him become more aware of what he was really feeling:

I have become much more awake in regard to [my] signals, and I find it easier to feel what goes on up in my head and in my body at large, really.

He then went on to describe how the awareness of his own body enabled him to relax when tension arose. The ability to discern different aspects of experience had proved useful for him:

Being able to isolate them a little bit from each other, knowing that those are thoughts and those are feelings and those are bodily sensations. I think that can be quite useful for my part, and enable me to get my feet back on the ground.

A few participants talked about becoming aware of patterns in their life that needed to be attended to. One woman described how her increase in awareness made it possible for her to learn about patterns of behaviour that triggered hypomanic states. Another described how she became aware of her own role in ongoing interpersonal difficulties as a result of slowing down and noticing her own thoughts, fears and expectations.

#### A more constructive way of relating to distress

This theme centred on experiences of being more accepting of oneself and the present situation, along with a willingness to explore what was happening instead of trying to “fix” things or be swept up in one’s own reactions. Some participants voiced an insight that struggling against symptoms runs the risk of merely increasing the distress, as illustrated by this statement:

Fighting against anxiety, that’s the wrong strategy. I’ve been fighting it a lot over a number of years. But that’s like quicksand; you just sink deeper into it.

This somewhat counter-intuitive way of relating to one’s troubles was elaborated by another participant, in response to a question about what she saw as important about acceptance:

That you don’t push away what comes up, but that you somehow greet it and meet it. That’s why meditating was so hard for me at first. I had incredible trouble meeting what was there. I like to look at it as a shoulder full of muscular tension that you have to massage away. It’s the same. Only that it feels like a long line inside you with a lot of knots that you have to pass through instead.

Still another participant noticed that in relations to his worries and rumination:

Perhaps you lose the hook you get caught on. You know, it [the topic that causes worry] is on one’s mind anyhow, so you might as well think about it and treat it differently from what you are used to and which often is self-perpetuating. And instead say ‘okay, I’m not fighting you, just be with me.

Some participants noted that accepting the presence of difficult thoughts and feelings reduced their anxiety to manageable proportions. Even a participant that felt that the course had not helped much to relieve her anxiety reported thinking that her anxiety was now “a bit more okay than it used to be.”

#### A more friendly relationship with myself

The present theme involved statements from a few participants indicating a more friendly and compassionate attitude towards themselves. This involved feeling entitled to just be who one is, without having to perform in specific ways or adjusting excessively to the expectations of others. One participant noted:

Just allowing oneself to be. When you’ve gotten used to performing all the time, that’s a welcome relief. To have a loving attitude towards oneself, that is a nice thought.

Another saw her self-view as having been implicated in her anxiety:

I think it’s at the bottom of a lot of my anxiety that I’m afraid what others might think about me all the time. That’s what’s at the heart of it. And then comes having to perform in all settings, both at work and in social gatherings, where I need to be good enough, and things like that. I feel that has eased down a bit, it’s not in me all the time. I manage to be a bit more myself, you know, and be a bit more relaxed and not needing to have things be in a certain way all the time.

#### Awareness in everyday activities

This fourth theme involved being present in everyday life in a new way, paying more concrete attention to sensations and moments that would formerly easily just have passed by. This entailed carrying out everyday activities such as eating, drinking, self-care, walking, or waiting with a greater degree of deliberate attention and focus on the experiences for their own sake. Two participants described how this newfound attitude had changed their experience of waiting, seeing waiting not as an absence of things happening but rather an opportunity to be with their own experience. One of these participants said this was part of a greater shift from him, from evaluating himself in terms of what he had accomplished to being more concerned with the quality of experience—an account in keeping with the theme of changing view of self.

Again, it is evident that the themes are interrelated. For instance, one participant noted that the emphasis on everyday awareness helped him realize how much time he was being lost in his own thoughts, and how this caused him to miss out on good experiences:

For example, I drove to work, and I usually drive by a lake, and then I saw the wind create ripples on the surface of the water. And the reason I noticed, was that the day before I’d been at the course and we’d been talking about noticing those kinds of things, right. I had an a-ha experience. I’ve stopped doing this, and I’ve started thinking instead, all those difficult thoughts of mine, instead of looking out of the window of my car and noticing those little things.

The participant went on to describe how focusing on visual aspects of the scenery makes his negative thoughts fade away, as he isn’t able to do both at once.

### Better—But not there yet

Despite the positive changes described, most participants did not see their problems as being fully solved after eight weeks of MBSR. Four of the sixteen participants reported still being significantly troubled by anxiety, and one additional participant did not feel that the course had led to any changes in her depressive symptoms. One told about unresolved issues with anger, and two highlighted their perceived vulnerability to stress as a continuing challenge.

Regardless of different accounts of change, all participants reported at least some benefit from the course. They felt they gained familiarity with a set of skills that require continued practice for full benefit. They conveyed that the approach had made sense to them, and the majority reported an expectation that there would be future benefits if they continued practicing. One of the participants still troubled by continuing anxiety put it like this:

There have been situations where I’ve had anxiety attacks, where it [practicing mindfulness] didn’t work as an emergency measure. And I don’t think that’s the purpose, either … you can’t, it won’t work, because they are so entrenched, those thoughts. But given time, it will change the pattern of how you think, how you handle things, I put my trust in that.

Among expected future benefits were increased ability to let go of thoughts, increased calm, and an ability to accept things as they are. Most participants indicated that they wished to keep up the mindfulness practice, but a number reported that this was challenging in the absence of a supportive group like the course had provided them with.

## Discussion

This study aimed to contribute to the understanding of how participants with anxiety disorders experience change when undergoing an 8-week MBSR course. Five themes and corresponding sub-themes were identified in the analysis of participants’ accounts: Something useful to do when anxiety appears, Feeling more at ease, Doing things my anxiety wouldn’t let me, Meeting what is there, and Better—but not there yet.

The first theme “Something useful to do when anxiety appears”, indicates that the MBSR course provided participants with strategies enabling them to shift their attention to a concrete focus or letting go of negative thinking. The descriptions of an attentional shift to a concrete focus such as the body or the breath is in line with theoretical descriptions of mindfulness as a skill that enables attending to the felt sense of experience, as opposed to cognitive evaluation or elaboration of what is happening. It may involve a “turning towards” experience regardless of its emotional valence, and an emphasis on processing the concrete details of physical sensations in the body as opposed to judging, ruminating about, or trying to eliminate internal experiences (Feldman & Kuyken, 2019). It is assumed that this type of present-centred awareness can be of value in that it may prevent psychological processes involved in anxiety from being triggered or escalated (Bishop et al., 2004; Roemer et al., 2009). It is possible that returning to the focus of the breath after noticing psychological symptoms of anxiety, may have reduced the feeling of threat associated with anxiety and the tendency to be aversely focused on physiological symptoms, which typically worsens negative affect (Mor & Winquist, 2002). This finding accords well with the finding of Allen et al. (2009) that participants in Mindfulness Based Cognitive Therapy for prevention of recurrent depression, pointed to the usefulness of “intentional, deliberate responses that shifted attention from a negative focus towards either a neutral or positive focus” (Allen et al., 2009, p. 419). Regulating anxiety through shifting the focus of attention to concrete bodily sensations can also be understood in terms of acquiring grounding techniques to handle difficult emotional states (Ogden & Fisher, 2015). A central clinical feature of anxiety disorders is the tendency for scarce attentional resources to be bound up in a hypervigilant scanning for cues of threat or danger (Bar-Haim et al., 2007). Even if many of the participants seem to utilize a present focus on bodily sensations or the breath to control their anxiety, they also seem to be less caught in their anxiety and have a better ability to self-regulate their attention

Being less bound up by anxiety is also reflected in the sub-theme “Letting go of thoughts”. By being able to identify and letting go of thoughts, participants felt less stuck in anxiety-reinforcing ruminative loops. Repetitive negative thinking is a common process across anxiety disorders (McLaughlin & Nolen-Hoeksema, 2011). Rumination is often focused on the present or past, while worry is a process of future-focused thinking characterized by overestimating negative outcomes and related tension and anxious apprehension (Barlow, 2002). Rumination and worry constitute forms of negative self-absorption (Ingram, 1990; Mor & Winquist, 2002; Nolen-Hoeksema et al., 2008) that prevents the individual from relating in an adaptive manner to current situations and challenges. Experiencing an improved ability to let go of thoughts, can be linked to theoretical descriptions of mindfulness as providing an increased meta-cognitive awareness, that is, the ability to perceive thoughts as thoughts as opposed to relating to them as literal truths about the self or the world (Corcoran & Segal, 2008). Studies indicate that mindfulness training may be beneficial particularly by reducing worry and rumination (Jain et al., 2007; Michalak et al., 2011; Van Aalderen et al., 2012). By being able to identify and letting go of thoughts, participants may also have to a lesser extent have tried to control or supress their thoughts, which usually increases distress (Abramowitz, Tolin & Street, 2001). Overall, the types of change described within the first theme seems to relate to handling well-known patterns within the cognitive domain characteristic of anxiety disorders: attentional biases to threat (Craske et al., 2009), aversive self-focused attention (Ingram, 1990; Mor & Winquist, 2002), and a tendency to catastrophic interpretations and repetitive negative thinking in the form of worry or rumination (McEvoy et al., 2013; McLaughlin & Nolen-Hoeksema, 2011).

The second theme, “Feeling more at ease” encompassed experiences of increased relaxation or decreased levels of stress or anxiety. For some participants, there seems to be a regulation of anxious emotions due to being able to prioritize tasks, doing one task at the time, and being less rushed when conducting various tasks. Others reported being more calm, relaxed or harmonious. While relaxation is not a goal per se in mindfulness exercises, it is often reported as an outcome of mindfulness training (Sevinc et al., 2018). It has also been established that these interventions are associated with decreased levels of psychological distress (Goldberg et al., 2018; Hofmann et al., 2010; Sevinc et al., 2018). Theoretical accounts of potential mechanisms involved, emphasize the paradoxical nature of mindfulness training: By allowing for experience to be as it is without trying to control or change it, the secondary reactions that would normally exacerbate suffering are diminished (Roemer et al., 2008). As a consequence, overall symptom levels could decrease despite this not being an explicit intention. The soothing effect of non-judgemental acceptance of experience can be understood as a form of meta-relaxation (Sevinc et al., 2018), that is an ability to relax through changing one’s relationship with inner experience. Overall, the types of change described within the second theme (“Feeling more at ease”) seems to imply improved strategies for emotion regulation, and for some, a general reduction in the level of anxiety and stress.

The third theme “Doing things my anxiety wouldn’t let me”, included sub-themes of breaking with patterns of avoidance, as well as being both more self-assertive and attuned in interpersonal communication. Both the decrease in anxiety and the ability to see anxiety as less threatening contributed to expanding participants’ repertoire of action. It is interesting that participants report being able to do things their anxiety had prevented, given that MBSR puts no emphasis on systematic exposure procedures. Nevertheless, the program contains a number of activities that can be seen as providing interoceptive exposure, helping participants relate differently to thoughts, feelings, and bodily sensations that had previously been avoided. In this sense, participants’ descriptions of relating differently to distress seems consistent with a decrease in experiential avoidance, that is, the tendency to try to change the form, frequency, and intensity of inner experience (Hayes et al., 2004). An interesting note to this third theme, is that some individuals with anxiety disorder seems to benefit from gaining a sense of agency and skill in regulating anxiety before they expose themselves to situations they have previously avoided. This interpretation is in line with A. Hjeltnes et al. (2018b) who found that participants with social anxiety who benefited from MBSR, reported the ability to use mindfulness exercises to approach unpleasant experiences. A strengthened belief in their own ability to handle anxiety seems to motivate them to then expose themselves to situations where they know anxiety can become intense. This may imply that there is not a necessary conflict between spending time in therapy gaining anxiety regulatory skills and direct exposure. It may even be the case that for some individuals with anxiety disorder, establishing a certain agency when it comes to handling anxiety by way of mindfulness practice, might benefit later exposure.

The third theme also included sub-theme of “Doing things differently in relationships”. It has been repeatedly found that anxiety disorders are associated with interpersonal fears and problems (Alden & Taylor, 2004; Eng & Heiberg, 2006; Hoffart et al., 2006). It is therefore interesting that a number of participants pointed to positive changes in their relationships, in the sense of greater assertiveness and clarity in communication. In some accounts this was linked to a decrease in anxiety, while in others it seemed that mindfulness exercises had given them a more precise sense of their own wishes, intentions, and boundaries. This latter finding is in line with what was reported by Allen et al. (2009) under the heading of “relationships”. Most participants in their study had developed an awareness of having automatically and habitually put other needs before their own, and experienced that the mindfulness course had “enabled them take better care of themselves with a sense of legitimacy and with reduced guilt” (Allen et al., 2009, p. 421). A study by Dekeyser et al. (2008) supports the notion that mindfulness interventions might impact relational skills. These authors found scores on a measure of mindfulness to be positively associated with expressing oneself in social situations, as well as with engagement in empathy. Also, Mace (2008) reported from an exploratory qualitative study that participants described a sense of being more sensitively attuned to others as a result of mindfulness practice. This is in line with findings from the present study with participants reporting an improved ability to listen to others. Overall, the types of change described within the third theme of this study seems to correspond to reduction of various forms of avoidance behaviours, another well-known characteristic of anxiety disorders.

In the fourth theme, “Meeting what is there”, there was an emphasis on letting go of the struggle and accepting present anxiety as it is, this includes being more able to stop and notice distress, relating to distress in more constructive ways, being more friendly with oneself, and being more aware of everyday activities. The capacity to pause resonates well with theoretical accounts of mindfulness as being opposed to operating on “automatic pilot”, and instead slowing down to enable more deliberate choices of action (Williams, 2010). This experience of change seems to most directly represent the assumed main path to change in mindfulness interventions, noticing and accepting own experiences and letting it be. Mindfulness and acceptance can be seen as modes of emotion regulation that are not based on the deliberate manipulation of the affective experience itself. Instead, it entails a shift from representational experience (an evaluative, instrumental mode of processing) to a more direct mode of experiencing (Williams, 2010). While anxiety entails a state where one`s attention is focused on scanning for threats from within or without, a mindful stance allows for experience to ebb and ﬂow more freely, without putting rigid demands on sensations, thoughts, or emotions. A mindful stance is also often characterized by a sense of care, warmth and kindness (self-compassion) towards oneself (Neff, 2003), which can also be seen as an emotion regulation strategy (Svendsen et al., 2016).

Overall, the types of change described within the fourth theme “Meeting what is there”, seems to reflect two types of change. First, an increase in emotional awareness and clarity through being able to stop and notice distress and where it is located in the body. Second, a type of change involving relating to themselves in less maladaptive and critical ways. Increased self-acceptance and kindness was however mentioned explicitly by only a few participants. We had expected this to be a more central feature in descriptions of outcome, but in the current sample changes in self-perception were more often described within the framework of greater self-assertion rather than kindness and compassion towards oneself.

A tension found in the data was between using mindfulness as a tool to regulate or get rid of anxiety, versus bringing forth greater acceptance of anxiety. Mindfulness can be described as a skill that allows mental and emotional states arise and pass without being prolonged by attempts to “fix” them. This skill is hypothesized to facilitate a more “decentred” perspective, where the person is less identified with the content of thoughts and feelings and more able to view them as transient phenomena that do not need to be acted upon. However, adhering to the exercises as techniques that can be applied to bring about change seemed to instal a sense of control and predictability that could aid participants in dealing with anxiety. These strategies express a wish on the part of the participants to remove anxiety, which is highly understandable, and was described as helpful. Reliance on mindfulness to get rid of their anxiety indicates that most participants had not fundamentally changed their relationship to experience towards a more radically accepting stance. The complex and counter-intuitive nature of mindfulness can take time to grasp. This might particularly be the case for individuals who have been suffering from anxiety for quite some time; wanting to be free from this pain is understandable. It is thus not surprising that a tension between fixing and letting be still remains in the narratives of a number of participants. It is also the case that the dialectical relationship between acceptance and change, or the modes of “being” and “doing”, are always intertwined in mindfulness work across diagnostic categories, as was also found by Allen et al. (2009) when investigating participants’ experience of a mindfulness-based group intervention for prevention of depressive relapse. Psychotherapy research indicates that therapeutic changes affect different domains progressively over time. Early change is primarily expressed in decreased symptom level, while changes in interpersonal functioning as well as in self-concept and self-experience typically take place in later phases (Howard et al., 1996; Kopta et al., 1994; Sembill et al., 2019). In light of this, it is perhaps not surprising that we find frequent descriptions of mindfulness as a method to decrease anxiety. After all, eight weeks is a relatively short time, and one would expect more fundamental changes in self-view to take longer to manifest. The fifth theme to emerge (“Better—but not there yet”) indicates that the participants themselves saw the MBSR course as a start, and that further and more comprehensive types of change could be expected if they were to keep up their practice. On the other hand, several of the participants described an increased ability to notice and express their own needs. Their improved assertiveness may reflect changes in underlying attitudes towards themselves and a heightened sense of self-worth.

### Reflexivity

How might the researchers’ grounding in the theory and practice of mindfulness and MABIs have affected the research process in the present study? The fact that the interviewers were familiar with the MBSR program content and intention unavoidably caused them to approach the interviews with certain preconceptions and a certain horizon of understanding. This might have the constructive potential of facilitating communication, as the interviewers could follow up the accounts by asking questions pertinent to the phenomenon of mindfulness training. However, investigators naïve to the phenomenon of mindfulness might have approached the interview situation differently, attending to other aspects of the participants’ experience.

As far as the analytic procedure is concerned, the intention was to identify themes and sub-themes from the material in a way that reflected the understanding and wording of the participants. If governed too much by our own theoretical understanding, it might be difficult to discover unexpected facets of the material. However, we did find it surprising that the tension between “fixing” and “letting be” was so manifest in the material. By accessing our own experience of mindfulness training, we were reminded how frequently a motivation to improve or perform of change things enters into the exercises, and how the practice is about acknowledging such motivation, while at the same time doing one’s best to return in a non-judgemental way to whatever is present in the moment (Kabat-Zinn, 1990). In this sense, our own experience helped us make sense of our findings.

### Scope and limitations

There are several important limitations in this study. First, as mentioned above, all interviewers in the present study was familiar with the ideas of mindfulness. In one respect, this could have been beneficial, as the understanding of ideas behind mindfulness interventions might have made it easier for the participants to explain their experiences. However, pre-existing knowledge of mindfulness might also have prevented interviewers from asking some more “naïve” questions that might uncover other participant experiences. Still, the fact that most participants did not mention acceptance and self-compassion in the foreground of their experience of change, might be interpreted as a sign that the interviewers did not direct the interviews in a biased way. In future studies it might still be of interest to combine interviewers with mindfulness experience and those without, to compare advantages and disadvantages.

Second, a limitation of the current study is that no formal evaluation of the adherence or competence level of the MBSR teacher was performed. A feature of mindfulness programmes is that systematic and sustained training in formal and informal mindfulness practices is central also for teachers of mindfulness programs (Crane et al., 2017). MBIs have an emphasis on acceptance and how people relate to their experiences. It is a possibility that mindfulness experience in teachers affects the extent to which the teacher can efficiently communicate basic principles such as acceptance and letting go. Without a formal evaluation of teacher competence, this theme cannot be investigated. In future studies, evaluation of teacher competence is highly encouraged.

Third, we unfortunately did not conduct interviews with those who did not complete the intervention. In retrospect, that would could have contributed to an understanding of their experiences leading to giving up on this particular treatment. Furthermore, as MBSR represents a broad set of strategies for dealing with internal experience, these may be more or less available depending on previous experience with psychotherapy. MBSR shares with cognitive therapy the perspective that perception and thought drive emotion and behaviour, and that if one changes one’s relationship to thought, one can change deeply ingrained self-destructive or maladaptive patterns of behaviour. One question is whether strategies of reappraising and modifying thought content learned in cognitive therapy would make it more or less complicated to practice mindfully observing and accepting thoughts no-matter thought-content. Likewise, whether previous experience with more formalized behavioural exposure procedures impact promoting a stance of openness, curiosity and acceptance of emotions. Unfortunately, we were not able to explore these questions based on our data.

### Implications for research and clinical practice

A possible implication of the findings in the present study is securing an even greater emphasis on the fact that the major goal of mindfulness-based interventions is getting to know oneself better and becoming softer and more playful and flexible, not to cultivate a perfected version of oneself. Meditation practice is not about trying to change oneself but befriending oneself and recognizing our tendencies to be reactive and stuck. Even as long-time practitioners of mindfulness, we repeatedly need to be reminded of this foundational attitude. One possible consequence of the finding that participants of 8 week courses in MBSR often struggle with basic concepts and attitude of acceptance, is an increased emphasis on this core ingredient of mindfulness-based programs in professional training and supervision.

## Conclusion

The aim of this study was to investigate experiences of change among individuals with anxiety disorders who participated in a clinical trial of MBSR. Our findings suggest that participants’ experience of change in MBSR correspond with areas known as transdiagnostic features of anxiety disorder: dysfunctional cognitive processes (including attentional biases), emotional dysregulation, avoidance behaviours, and maladaptive self- relatedness. This supports MBSR as a transdiagnostic approach to the treatment of anxiety disorders. Interestingly, it was not uncommon for participants to speak of mindfulness both as a tool to “fix” anxiety, and as bringing about a more fundamental change towards acceptance of their anxiety. Many used the acceptance of present moment experience for instrumental purposes, and seemed to find relief from their anxiety and a sense of agency in so doing. A few reported more radical acceptance of their anxiety, as well as increased self-acceptance and kindness towards themselves. We hypothesize that pervasive acceptance is an aspect of mindfulness training which may emerge later than initial symptom reduction.
